# A Study of Nuclear Factor-Kappa B1 Gene Polymorphism Types in Schizophrenia Patients and Their Correlation With Disease Severity

**DOI:** 10.7759/cureus.24401

**Published:** 2022-04-22

**Authors:** Susree A Swain, Pratikhya Sarangi, Roma Rattan, Pratima K Sahu, Andrew A Lamare

**Affiliations:** 1 Biochemistry, SCB Medical College, Cuttack, IND; 2 Biochemistry, SCB Medical College and Hospital, Cuttack, IND

**Keywords:** schizophrenia, nf-κb gene polymorphism, mental health, inflammation, genotype

## Abstract

Background and objective

Schizophrenia is a chronic mental illness that is associated with multifactorial causation, but the greatest risk factor is a positive family history. Previous studies have suggested that proinflammatory cytokines and acute-phase proteins increase and modulate the severity of symptoms of schizophrenia. The inflammatory milieu in these patients has been found to be controlled by the transcription factor nuclear factor-kappa B1 (NF-κB1) in the inflammatory cells. In this study, we aimed to examine the correlation between polymorphism of the NF-κB1 gene and the severity of disease symptoms in schizophrenia patients.

Materials and methods

This was a case-control study conducted on 90 diagnosed cases of schizophrenia patients with 90 matched healthy volunteers as controls. DNA was extracted from EDTA blood samples and PCR was run, and the study of the NF-κB1 gene polymorphism rs28362691 (-94 ATTG ins/del) was performed by using restriction fragment length polymorphism (RFLP).

Results

We observed the ins/ins genotype (78%) to be more prevalent among the study population. The del/del and ins/del genotypes were seen in 6.7% and 14.4% of schizophrenic patients respectively. The insertion allele was seen more than the deletion allele. Pearson’s correlation analysis showed a significant positive correlation between NF-κB1 levels and disease severity with an r-value of 0.471 and a p-value of 0.027.

Conclusion

We found that in schizophrenia patients, the insertion allele was higher than the deletion allele and the ins/ins genotype was higher in frequency than the del/del and ins/del genotypes. There was a strong positive association between the insertion genotype and the severity of disease symptoms in schizophrenia patients.

## Introduction

Schizophrenia is a chronic, severe psychiatric illness with a heterogeneous array of symptoms. It is characterized by profound disruptions of thought, which affect language, perception, and sense of self. It often includes positive psychotic experiences, such as hearing voices or delusions, hallucinations, i.e., false perceptions, disorganized behavior, disorganized speech, and mental apathy [1]. It is estimated to affect approximately 24 million people or one in 300 people (0.32%) worldwide [1]. The age of onset is most commonly during late adolescence and the twenties and men are more commonly affected than women [1]. Studies on the prevalence of schizophrenia in India have reported rates between 1.5 and 2.5 per 1000 population. Incidence studies are scarce, with estimates of an annual incidence of 0.35-0.38 per 1000 in urban populations and 0.44 per 1000 among the rural population [2,3].

Schizophrenia has been classically considered to be multifactorial in origin. The pathogenesis of the disease has been attributed in recent years to an interplay between genetics and environmental conditions [4]. Positive family history involving first-degree relatives of the patients is one of the greatest risk factors. The incidence has been reported to be around 6.5% in first-degree relatives of patients [5], and it rises to more than 40% in monozygotic twins of affected people [1]. The diagnosis of schizophrenia is made according to the International Classification of Diseases, 10th revision (ICD-10), i.e., the ICD-10 Classification of Mental and Behavioral Disorders by the WHO, code F20 (for schizophrenia), from F20.0-F20.9 [6]. The criteria include the following characteristics.

ICD-10 diagnostic criteria for schizophrenia [7]

At least one of the following features is present most of the time for a month

• Thought echo, insertion or withdrawal, or thought broadcast

• Delusions of control pertaining to body parts, actions, or sensations

• Delusional perception

• Hallucinatory voices giving a running commentary, discussing the patient, or coming from some part of the patient's body

• Persistent bizarre or culturally inappropriate delusions

Or at least two of the following symptoms are present most of the time for a month

• Persistent daily hallucinations accompanied by delusions

• Incoherent or irrelevant speech

• Catatonic behavior such as stupor or posturing

• Negative symptoms such as marked apathy and blunted or incongruous mood.

The Positive and Negative Syndrome Scale (PANSS) is a medical scale used for measuring symptom severity of patients with schizophrenia. It is widely used in the study of antipsychotic therapy [7]. Schizophrenia has been considered to be a polygenic disorder. A large number of genetic variants have been associated with the pathogenetic mechanism of the disease. Among them, the nuclear factor-kappa B (NF-kB) gene has been studied extensively [8]. Studies have suggested that proinflammatory cytokines and acute-phase proteins increase and modulate the severity of the symptoms of schizophrenia. The inflammatory milieu is far largely controlled by the transcription factor nuclear-factor kappa B1 (NF-κB1) inside the neuronal cells [9]. The transcription factor NF-κB1 was discovered in 1986 and it is a nuclear factor that binds to the enhancer element of the immunoglobulin kappa light chain of the activated B cells [10]. The NF-κB family in mammals has been identified to consist of five members: p65 (RelA), RelB, c-Rel, NF-κB1, and NF-κB2. NF-κB1 and NF-κB2 are synthesized as premature (p105 and p100) and then proteolytically cleaved into factors p50 and p52 [11]. All these five transcription factors can associate with each other, thereby forming homo or heterodimer units.

Thus, the NF-κB1 signaling pathway has been recognized as an important regulator of the growth and morphology of neural processes in the developing and mature nervous system. The primary mechanism for regulating NF-κB is through inhibitory IκB proteins (IκB, inhibitor of NF-κB1), and the kinase that phosphorylates IκBs, namely, the IκB kinase (IKK) complex [12]. NF-κB1 remains inactivated in the cytoplasm by noncovalent interaction with inhibitory proteins (IκBs). By external signals, inhibitor proteins are phosphorylated and degraded, and NF-κB1 gets activated and translocated to the nucleus to start expression in target genes [12].

Recent studies have suggested the overactivity of both arms of the NF-κB activation pathway in the cortex of people with schizophrenia. The transcription factor NF-κB is a critical regulator of immune responses and controls the expression of various proinflammatory cytokines and acute-phase proteins that are increased in the brain in people with schizophrenia [13]. Several variations have been identified in the NF-κB1 gene, including rs72696119 (C>G), rs28362491 (-94ins/del ATTG), rs4648068 (A>G), and rs12509517 (G>C). One of the single nucleotide polymorphisms (SNP) in the promoter region of NF-κB1 has been observed to affect nuclear protein binding and thus gene transcription [14]. rs28362491 polymorphism is a 4 bp ATTG insertion/deletion variation in -94 bp of NF-κB1 promoter [15]. This modification occurs within the promoter region of the NFκB1 gene, which potentially affects the transcription of the gene and the function of NF-κB protein, sequentially leading to a loss of binding capacity to nuclear proteins and reduced promoter activity [11].

Studies on NF-κB gene polymorphism and its relation to schizophrenia have been of recent origin. Studies are scarce from this part of the country on what the different polymorphisms of this -94 bp of NF-κB1 promotor region are and how they are associated with schizophrenia. In light of this, this study was undertaken to find a correlation between the polymorphism of the NF-κB1 gene and the severity of disease symptoms in schizophrenia patients.

## Materials and methods

Study design

This case-control study was conducted at the Post Graduate Department of Biochemistry, Molecular and Genomics Laboratory in collaboration with the Multidisciplinary Research Unit (MRU) and Mental Health Institute, SCB Medical College and Hospital in Cuttack, Odisha, India. The study comprised 180 samples, consisting of 90 cases (the study group) and an equal number of matched healthy volunteers as controls (the control group). The study group comprised patients with schizophrenia attending the Mental Health Institute at the SCB Medical College and Hospital, and both groups were classified based on the ICD-10 criteria.

Inclusion criteria

The study group included newly diagnosed cases of schizophrenia patients aged 18-55 years as per ICD-10, of either gender, attending the Mental Health Institute OPD at the SCB Medical College and Hospital. Healthy subjects aged 18-55 years of both genders with normal intelligence, who were physically stable without any physical disease, no history of head injury, and without neurological diseases were included in the control group.

Exclusion criteria

Old cases of schizophrenia, patients with a history of substance abuse or drug dependence except for nicotine, terminally ill patients, patients with any space-occupying lesions and other organic diseases of the brain like dementia, and those with other comorbid conditions like obesity and diabetes mellitus were excluded.

The demographic data were collected, which included age, gender, weight, height, BMI, and residence, and data related to disease severity (according to PANSS) were obtained. The disease severity was assessed based on the PANSS through a brief interview, requiring the patients to spend 45-50 minutes on a questionnaire. After that, 5 ml of venous blood was collected and all the routine biochemical investigations like random blood glucose, renal function tests, and liver function tests were done. Blood samples of 2 ml were collected in blood collection tubes with EDTA, and DNA was extracted from EDTA blood sample using the QIAGEN DNA Mini kit; DNA purity was checked with a NanoDrop™ spectrophotometer. The extracted DNA was then subjected to PCR using a GeNet Bio kit and run-in thermocycler from the Applied Biosystems (Applied Biosystems, Waltham, MA); 2% agarose gel electrophoresis was run for all post-PCR products (quality and quantity assessment) and the bands were visualized in the Bio-Rad Gel documentation system. The NF-κB gene polymorphism study of promoter site rs28362691 (-94 ATTG ins/del) was performed by using restriction fragment length polymorphism (RFLP) using the PflMI restriction enzyme.

Statistical analysis

The statistical analysis of data was performed using the SPSS Statistics version 28 (IBM, Armonk, NY). Results were expressed as means and standard deviations (SD) for continuous variables and as percentages for categorical variables. Data were compared using an unpaired student's t-test and a one-way analysis of variance (ANOVA) post-hoc Turkey honestly significant difference (HSD) test. Pearson’s correlation coefficient was used, and a p-value <0.05 was considered statistically significant.

## Results

Table 1 shows the various demographic parameters including age group, gender distribution, and geographical distribution among the study population. We observed that a majority of the patients were 35-47 years in age (43%). Regarding gender distribution, the male gender was more predominant than females (53.3% males and 46.7% females). In terms of geographical distribution, the urban population was more affected than the rural. In the comparison of anthropometric profiles between cases and controls, no significant difference was found.

**Table 1:** Demographic characteristics and anthropometric data of the study participants BMI: body mass index; SD: standard deviation; SBP: systolic blood pressure; DBP: diastolic blood pressure

Variables	Values		
Age (years)	Number of individuals (%)		
22-34	58 (32%)		
35-47	77 (43%)		
48-55	45 (25%)		
Gender	Number of individuals (%) (n=180)		
Male	96 (53.30)		
Female	84 (46.70)		
Geographical distribution			
Urban	113 (62.8%)		
Rural	67 (37.2%)		
Anthropometric parameters	Cases (n=90), mean ±SD	Controls (n=90), mean ±SD	P-value
Weight (kg)	54.07 ±4.484	54.07 ±4.484	1
Height (meters)	1.59 ±0.040	1.59 ±0.040	1
BMI (kg/m^2^)	21.22 ±1.86	21.05 ±1.83	0.544
Blood pressure			
SBP	120.80 ±6.20	120.07 ±4.339	0.36
DBP	71.87 ±5.89	72.07 ±5.62	0.816

Table 2 shows the comparison of routine biochemical parameters between cases and controls. All data were represented as mean ±SD and were compared by unpaired student’s t-test. A p-value ≤0.05 was considered significant. No significant difference in routine biochemical parameters was observed between the schizophrenia patient group and healthy volunteers.

**Table 2:** Comparison of routine biochemical parameters between schizophrenia patients and healthy volunteers SD: standard deviation; RBS: random blood sugar; SGOT: serum glutamic-oxaloacetic transaminase; SGPT: serum glutamic-pyruvic transaminase

Parameters	Healthy volunteers (n=90), mean ±SD	Schizophrenia patients (n=90), mean ±SD	P-value
RBS	149.90 ±67.90	177.91 ±30.28	0.145
Renal function tests			
Serum urea (mg/dl)	32.48 ±19.34	26.63 ±9.92	0.32
Serum creatinine (mg/dl)	1.1 ±0.49	0.87 ±0.43	0.201
Liver function tests			
Serum total bilirubin (mg/dl)	0.85 ±0.44	0.92 ±0.46	0.304
Serum direct bilirubin (mg/dl)	0.39 ±0.20	0.43 ±0.23	0.268
SGOT (IU/L)	28.37 ±13.32	31.10 ±19.34	0.273
SGPT (IU/L)	75.93 ±25.94	36.06 ±36.19	0.101
Serum alkaline phosphatase (IU/L)	245.92 ±93.04	223.44 ±78.30	0.081

Table 3 shows the frequency of various genotypes of NF-κB1 -94ins/del ATTG promoter polymorphism. We observed that the ins/ins genotype (78%) was more prevalent among the study population. The del/del and ins/del genotypes were seen in 6.7% and 14.4% of schizophrenic patients respectively. The insertion allele was seen more than the deletion allele: 86.1% and 87.2% in cases and controls respectively.

**Table 3:** Comparison of NF-κB1 -94ins/del ATTG promoter polymorphism genotypes between the groups NF-κB1: nuclear factor-kappa B1

	Cases (n=90), n (%)	Controls (n=90), n (%)
ins/ins	71 (78.9%)	77 (85.6%)
del/del	6 (6.7%)	10 (11.1%)
ins/del	13 (14.4%)	3 (3.3%)
ins allele	155 (86.1%)	157 (87.2%)
del allele	25 (13.9%)	23 (12.8%)

**Figure 1:**
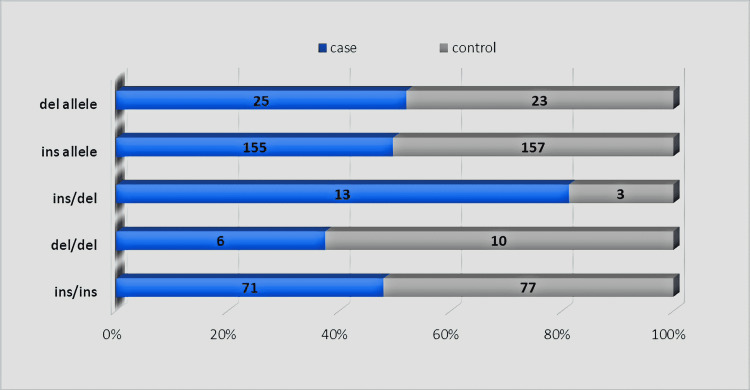
Frequency of various NF-κB1 genotypes among the study population NF-κB1: nuclear factor-kappa B1

Figure 1 shows the bar diagram depicting the frequency of NF-κB1 polymorphism genotypes among the study population. Blue color codes are for cases and gray color codes represent controls.

**Figure 2:**
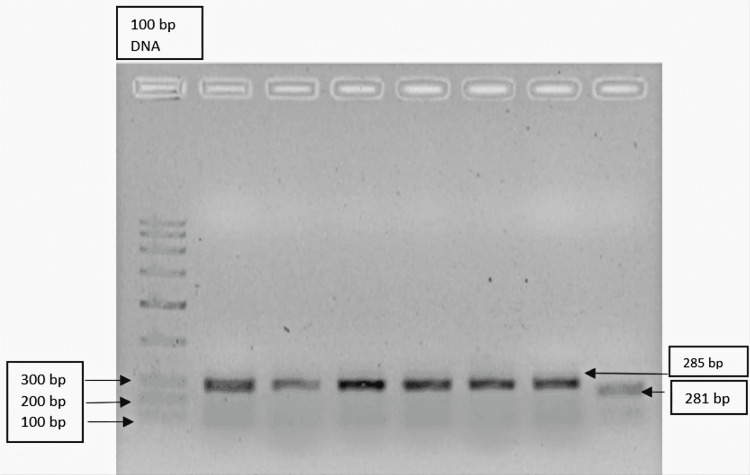
Agarose gel picture showing post-PCR DNA bands

Figure 2 depicts the agarose gel picture showing post-PCR DNA bands of NF-κB1 gene, LANE 1: 100 bp LADDER, LANE 2-7: 281 bp bands, and LANE 8: 240 bp band.

**Figure 3:**
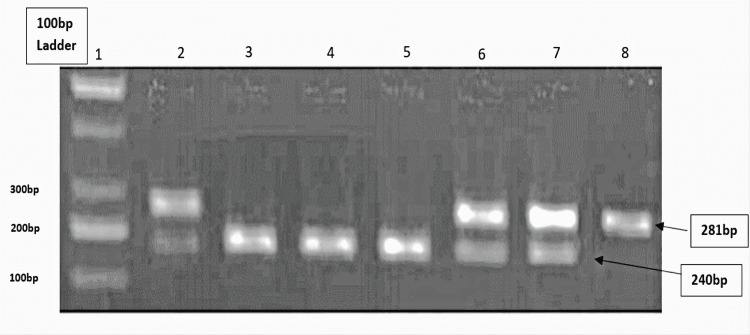
Agarose gel picture showing RFLP bands RFLP: restriction fragment length polymorphism

Figure 3 illustrates the agarose gel picture showing RFLP bands of the NF-κB1 gene with rs28362491 using PflMI restriction enzyme, LANE 1: 100 bp LADDER, LANE 2,6 and 7 are ins/del ATTG; LANE 3,4 and 5 are ins/ins ATTG, and LANE 8 is del/del ATTG genotype.

Table 4 shows the Pearson’s correlation analysis between NF-κB1 levels and disease severity. A significant positive correlation was observed with an r-value of 0.471 and a p-value of 0.027.

**Table 4:** Correlation between NF-κB1 gene polymorphism and the severity of the disease among schizophrenia patients NF-κB1: nuclear factor-kappa B1

NF-κB1 gene polymorphism insertion allele genotype vs. disease severity	r-value	P-value
	0.471	0.027

## Discussion

Our study was undertaken at a tertiary care hospital and it was a retrospective case-control study where controls were healthy individuals and cases consisted of patients with schizophrenia. Both genetic and environmental factors contribute to the neuroinflammation development of schizophrenia. Recent studies have associated schizophrenia with inflammation and genetic predisposition [16]. The level of NF-κB1 activity is regulated at the mRNA level, thus NF-κB1 is a transcription factor that is regulated by a transcriptional protein within the cell. The NF-κB1 signaling pathway is constitutively expressed in the neural cells where it regulates the expression of certain genes involved in cell survival, memory, as well as synaptic plasticity [17]. Dysregulation of the NF-κB1 pathway has been associated with the pathogenesis of schizophrenia, making NF-κB a suitable marker to ascertain the cause of neuroimmune dysregulation in schizophrenia [17]. Variations in the NF-kB1 binding sequences in the promoter region of the gene encoding the cytokines and inflammatory molecules are one of the major attributes of the disease severity and progression in schizophrenia patients [17].

We found that schizophrenia was more prevalent among the male study participants (53%) than females (46.7%) (Table 1). The gender variation seen in our study is similar to that in various other epidemiological studies on schizophrenia [18]. A two-year follow-up study by Usall et al. in 2003 examined the gender differences in schizophrenia and observed that the disease is more severe in males and the frequency and duration of hospitalization were also higher in men compared to females [18]. Women seem to have two peaks in the age of onset of the disease: the first after menarche and the second once they are over 45 years, which could be explained by the reduction of estrogens after menopause according to the estrogenic hypothesis of schizophrenia [18]. Symptoms of schizophrenia are gender dimorphic, with half of the studies showing more depressive symptoms in women and more negative symptoms in men, and the other half showing no such difference. The high prevalence of substance abuse in men with schizophrenia may contribute both to an earlier onset and a more severe course in men as compared to women [19,20]. In recent studies conducted by Li et al, it has been shown that sex hormones have a regulating effect on mental functions, suggesting that sex chromosomes, XX or XY, may have roles in the neurodevelopment and contribute sex-specific cognitive functions.

In this study, we observed that the majority of the participants were 35-47 years of age, as shown in Table 1. The studies conducted by Gogtay et al. suggested that with many major neuropsychiatric illnesses, the typical age of onset is late adolescence. In schizophrenia, the period of onset is usually late adolescence or early twenties, while the age of onset is 25-35 years in females. Adolescence is a critical period in terms of neural development, and the relationship between normal brain maturation and the onset of psychopathology in this age group is pertinent [21]. In studies conducted by Li et al., it has been reported that men usually develop the illness at the age of 18-25 years, while in women, the mean age of onset is 25-35 years. The onset of schizophrenia depends on both structural and functional abnormalities in the brain, which are affected by events from the prenatal period till adulthood to complex heritable genetic disorders [20].

In our study, we found that the majority of the schizophrenia patients (63%) resided in urban areas while 37% were from rural areas (Table 1). Several studies and a meta-analysis conducted by Vassos et al. support the association of exposure to an urban environment with the development of schizophrenia. City life predisposes individuals to schizophrenia due to sociodemographic structures, neighborhood effects, social fragmentation, and deprivation [21,22]. We observed no significant difference in routine biochemical parameters between schizophrenia patients and healthy volunteers (Table 2).

We evaluated the possibility of genetic variants affecting the disease causation and severity and found a variation in the RFLP of the NF-kB1 transcription factor with rs28362691 sequence of (-94ins/delATTG). Table 3 shows the frequency of various genotypes of the NF-kB1 -94ins/delATTG promoter region of both groups. We observed the ins/ins genotype (78%) to be more prevalent among the study population. The del/del and ins/del genotypes were seen in 6.7% and 14.4% of schizophrenic patients respectively. The ins allele was seen more than the del allele, i.e., 86.1% and 87.2% in cases and controls respectively.

Figure 2 shows the agarose gel electrophoresis of the post-PCR DNA bands of the NF-kB1 gene. In our study, we observed that the insertion allele was higher than the deletion allele and the ins/ins genotype was higher in frequency than the del/del and ins/del genotype. This is in line with studies conducted by Liou et al. [14]. They suggest that NF-kB1 is a key transcriptional factor in the regulation of the expression of many inflammatory factors, such as cytokines, chemokines, and adhesion molecules. They have demonstrated that cerebrospinal fluid (CSF) and plasma of schizophrenic patients had abnormal levels of cytokines, and the aberrations were especially more pronounced in treatment-refractory schizophrenia cases. Song et al. also found that the elevated level of cytokine in first-episode schizophrenic patients was associated with the activation of NF-kB1 [10]. One common SNP variant rs28362691 (-94ins/delATTG) was identified from sequencing the NF-kB1 gene and it was found to be associated with schizophrenia. The promoter assay showed that the NF-kB1 promoter with the -94delATTG allele had a lower promoter activity in comparison with the -94insATTG allele. This implies that the -94delATTG allele may result in lower expression of NF-kB1. Changes in NF-kB1 expression could alter the level of p105 and induce divergent dimeric combinations of NF-kB1, which can lead to various inflammatory cytokine dysregulations and also hamper the effects of antipsychotic treatment [15].

Similarly, the findings of a cross-sectional study conducted by Senol Tuncay et al. reported that a difference existed in ethnic populations with respect to genotypes and allele frequencies for the NF-kB1 -94ins/del ATTG polymorphism and for the NF-kB1 30 UTR A--->G polymorphism. Our findings are similar to those of a study among the Chinese population. Our study followed the Hardy-Weinberg equilibrium for this locus [23].

Figure 3 shows an agarose gel picture of post-RFLP DNA bands using the restriction enzyme PfIMI.

The early onset of schizophrenia has been proposed to be associated with a more severe form of the disease, with a potentially poorer prognosis and more neurodevelopmental insults [24]. Our study showed that the Pearson's correlation analysis between the NF-kB1 gene ins allele genotype and disease severity in schizophrenia patients had a significant positive correlation with an r-value of 0.471 and a p-value of 0.027 (Table 4).

The findings of our study can be used for disease monitoring since the brain tissue is inaccessible but can only be studied in postmortem cases; hence, the findings of our study can serve as biomarkers. In our study, we found a significant positive correlation between the NF-kB1 gene polymorphism and the severity of the disease. In the study conducted by Song et al., it has been found that NF-kB1 is sensitive to many factors such as illness and cytokines. In various other studies, it was observed that the NF-kB1 activation and its mRNA expression were excessively increased in schizophrenia patients compared with healthy subjects, which may be attributed to the increased cytokines. NF-kB1 in cells is activated in the acute state of schizophrenia, and the activated NF-kB1 moves to the nucleus and binds to kB sites in target genes that rapidly induce transcription, and hence cytokines can activate NF-kB and further inflammatory process, thereby creating a vicious cycle. This may be helpful in improving the treatment and monitoring the severity of the disease [25]. However, in our study, we found that the NF-kB1 gene polymorphism is highly significant in mild forms of the disease.

Limitations of the study

This study has a few limitations. Primarily, the study had a small sample size. Secondly, there were fewer individuals aged between 48 and 55 years compared to other age groups. Also, genetic markers were studied in the DNA extracted from WBCs of the blood and not from brain tissues. Finally, as it was a cross-sectional study, follow-up of the patients could not be done.

## Conclusions

The results of this study suggest that middle-aged men were more affected as compared to women, and there was a preponderance of urban residents compared to rural dwellers. In the schizophrenia group, the genotyping study of the promoter region of the NFκB1 gene found that the insertion allele was higher than the deletion allele and the ins/ins genotype was higher in frequency than the del/del and ins/del genotypes. Based on our findings, we propose that a genotyping study of the NF-κB1 gene could provide useful study parameters in schizophrenia patients.

## References

[1] Lewis DA, Lieberman JA (2000). Catching up on schizophrenia: natural history and neurobiology. Neuron.

[2] Charlson FJ, Ferrari AJ, Santomauro DF (2018). Global epidemiology and burden of schizophrenia: findings from the Global Burden of Disease Study 2016. Schizophr Bull.

[3] Cardno AG, Marshall EJ, Coid B (1999). Heritability estimates for psychotic disorders: the Maudsley twin psychosis series. Arch Gen Psychiatry.

[4] Schizophrenia Working Group of the Psychiatric Genomics Consortium (2014). Biological insights from 108 schizophrenia-associated genetic loci. Nature.

[5] Kendler KS, McGuire M, Gruenberg AM, O'Hare A, Spellman M, Walsh D (1993). The Roscommon Family Study. I. Methods, diagnosis of probands, and risk of schizophrenia in relatives. Arch Gen Psychiatry.

[6] (2022). ICD-10 Classification of Mental and Behavioral Disorders, WHO, Geneva, Diagnostic criteria for research. https://apps.who.int/iris/handle/10665/37958.

[7] Picchioni MM, Murray RM (2007). Schizophrenia. BMJ.

[8] Smeland OB, Frei O, Dale AM, Andreassen OA (2020). The polygenic architecture of schizophrenia - rethinking pathogenesis and nosology. Nat Rev Neurol.

[9] Hoesel B, Schmid JA (2013). The complexity of NF-κB signaling in inflammation and cancer. Mol Cancer.

[10] Song XQ, Lv LX, Li WQ, Hao YH, Zhao JP (2009). The interaction of nuclear factor-kappa B and cytokines is associated with schizophrenia. Biol Psychiatry.

[11] Oeckinghaus A, Ghosh S (2009). The NF-kappaB family of transcription factors and its regulation. Cold Spring Harb Perspect Biol.

[12] Senol Tuncay S, Okyay P, Bardakci F (2010). Identification of NF-kappaB1 and NF-kappaBIAlpha polymorphisms using PCR-RFLP assay in a Turkish population. Biochem Genet.

[13] Murphy CE, Lawther AJ, Webster MJ (2020). Nuclear factor kappa B activation appears weaker in schizophrenia patients with high brain cytokines than in non-schizophrenic controls with high brain cytokines. J Neuroinflammation.

[14] Liou YJ, Wang HH, Lee MT (2012). Genome-wide association study of treatment refractory schizophrenia in Han Chinese. PLoS One.

[15] Roussos P, Katsel P, Davis KL (2013). Convergent findings for abnormalities of the NF-κB signaling pathway in schizophrenia. Neuropsychopharmacology.

[16] Guttridge DC, Albanese C, Reuther JY, Pestell RG, Baldwin AS Jr (1999). NF-kappaB controls cell growth and differentiation through transcriptional regulation of cyclin D1. Mol Cell Biol.

[17] Murphy CE, Walker AK, Weickert CS (2021). Neuroinflammation in schizophrenia: the role of nuclear factor kappa B. Transl Psychiatry.

[18] Gutierrez H, Davies AM (2011). Regulation of neural process growth, elaboration and structural plasticity by NF-κB. Trends Neurosci.

[19] Ochoa S, Usall J, Cobo J, Labad X, Kulkarni J (2012). Gender differences in schizophrenia and first-episode psychosis: a comprehensive literature review. Schizophr Res Treatment.

[20] Sommer IE, Tiihonen J, van Mourik A, Tanskanen A, Taipale H (2020). The clinical course of schizophrenia in women and men-a nation-wide cohort study. NPJ Schizophr.

[21] Gogtay N, Vyas NS, Testa R, Wood SJ, Pantelis C (2011). Age of onset of schizophrenia: perspectives from structural neuroimaging studies. Schizophr Bull.

[22] Vassos E, Pedersen CB, Murray RM, Collier DA, Lewis CM (2012). Meta-analysis of the association of urbanicity with schizophrenia. Schizophr Bull.

[23] Zammit S, Lewis G, Rasbash J, Dalman C, Gustafsson JE, Allebeck P (2010). Individuals, schools, and neighborhood: a multilevel longitudinal study of variation in incidence of psychotic disorders. Arch Gen Psychiatry.

[24] Melas PA, Rogdaki M, Ösby U, Schalling M, Lavebratt C, Ekström TJ (2012). Epigenetic aberrations in leukocytes of patients with schizophrenia: association of global DNA methylation with antipsychotic drug treatment and disease onset. FASEB J.

[25] Wan L, Wei J (2021). Early-onset schizophrenia: a special phenotype of the disease characterized by increased MTHFR polymorphisms and aggravating symptoms. Neuropsychiatr Dis Treat.

